# Identification of the potentiating mutations and synergistic epistasis that enabled the evolution of inter-species cooperation

**DOI:** 10.1371/journal.pone.0174345

**Published:** 2017-05-11

**Authors:** Sarah M. Douglas, Lon M. Chubiz, William R. Harcombe, Christopher J. Marx

**Affiliations:** 1 Department of Molecular and Cellular Biology, Harvard University, Cambridge, Massachusetts, United States of America; 2 Department of Organismic and Evolutionary Biology, Harvard University, Cambridge, Massachusetts, United States of America; 3 Department of Biology, University of Missouri-St. Louis, St. Louis, Missouri, United States of America; 4 Department of Ecology, Evolution, and Behavior, University of Minnesota, St. Paul, Minnesota, United States of America; 5 Department of Biological Sciences, University of Idaho, Moscow, Idaho, United States of America; 6 Institute for Bioinformatics and Evolutionary Studies, University of Idaho, Moscow, Idaho, United States of America; 7 Center for Modeling Complex Interactions, University of Idaho, Moscow, Idaho, United States of America; Universiteit Leiden, NETHERLANDS

## Abstract

Microbes often engage in cooperation through releasing biosynthetic compounds required by other species to grow. Given that production of costly biosynthetic metabolites is generally subjected to multiple layers of negative feedback, single mutations may frequently be insufficient to generate cooperative phenotypes. Synergistic epistatic interactions between multiple coordinated changes may thus often underlie the evolution of cooperation through overproduction of metabolites. To test the importance of synergistic mutations in cooperation we used an engineered bacterial consortium of an *Escherichia coli* methionine auxotroph and *Salmonella enterica*. *S*. *enterica* relies on carbon by-products from *E*. *coli* if lactose is the only carbon source. Directly selecting wild-type *S*. *enterica* in an environment that favored cooperation through secretion of methionine only once led to a methionine producer, and this producer both took a long time to emerge and was not very effective at cooperating. On the other hand, when an initial selection for resistance of *S*. *enterica* to a toxic methionine analog, ethionine, was used, subsequent selection for cooperation with *E*. *coli* was rapid, and the resulting double mutants were much more effective at cooperation. We found that potentiating mutations in *metJ* increase expression of *metA*, which encodes the first step of methionine biosynthesis. This increase in expression is required for the previously identified actualizing mutations in *metA* to generate cooperation. This work highlights that where biosynthesis of metabolites involves multiple layers of regulation, significant secretion of those metabolites may require multiple mutations, thereby constraining the evolution of cooperation.

## Introduction

Resolving the genetic and mechanistic bases of complex biological behaviors within or between cells remains a central challenge in the post-genomic era. The rare and delayed emergence of citrate use in experimentally evolved *Escherichia coli* has become a classic example of this challenge[1–4]. This work has pointed to the key role of “potentiation”, an initial mutation(s) that permits evolution of “actualization” through a subsequent mutation that fully allows the complex trait to emerge. Similarly, the early stages of inter-species cooperation may also tend to require multiple mutations. If multiple mutations are required to permit effective exchange of nutrients, how can this trait emerge? There are now many examples of metabolic exchange in natural microbial systems [5–7], yet the evolutionary events facilitating cooperation remain unclear. Synthetic systems have begun to provide clearer insights into the underlying mutations and mechanisms required for inter-species cooperation[8–19], but several questions remain. Namely, how do the ordering and functional impact of adaptive mutations impact the emergence of the cooperative phenotype?

The need for a potentiating mutation to precede an actualizing mutation is an example of epistasis. Epistasis describes when the phenotypic effect of a gene depends upon the genotype at one or more other loci[20]. In the experimental evolution of microbes, whereby evolution can be replayed from the same starting point, it has been commonly found that beneficial mutations generically provide proportionally less advantage when combined than expected from their individual effects, i.e., antagonistic epistasis (also known as diminishing returns) on fitter backgrounds[21–26], although counter-examples of synergistic epistasis have been shown[27]. The scenario of potentiating and actualizing mutations is a strong form of synergistic epistasis. Epistasis can greatly affect adaptation, from slowing the pace of fitness increase [28–29], to constraining the evolutionary trajectory of a population[30–32]. The requirement of potentiating and actualizing mutations to generate a novel phenotype is yet another example of the importance of epistasis in evolutionary processes.

**Fig 1:**
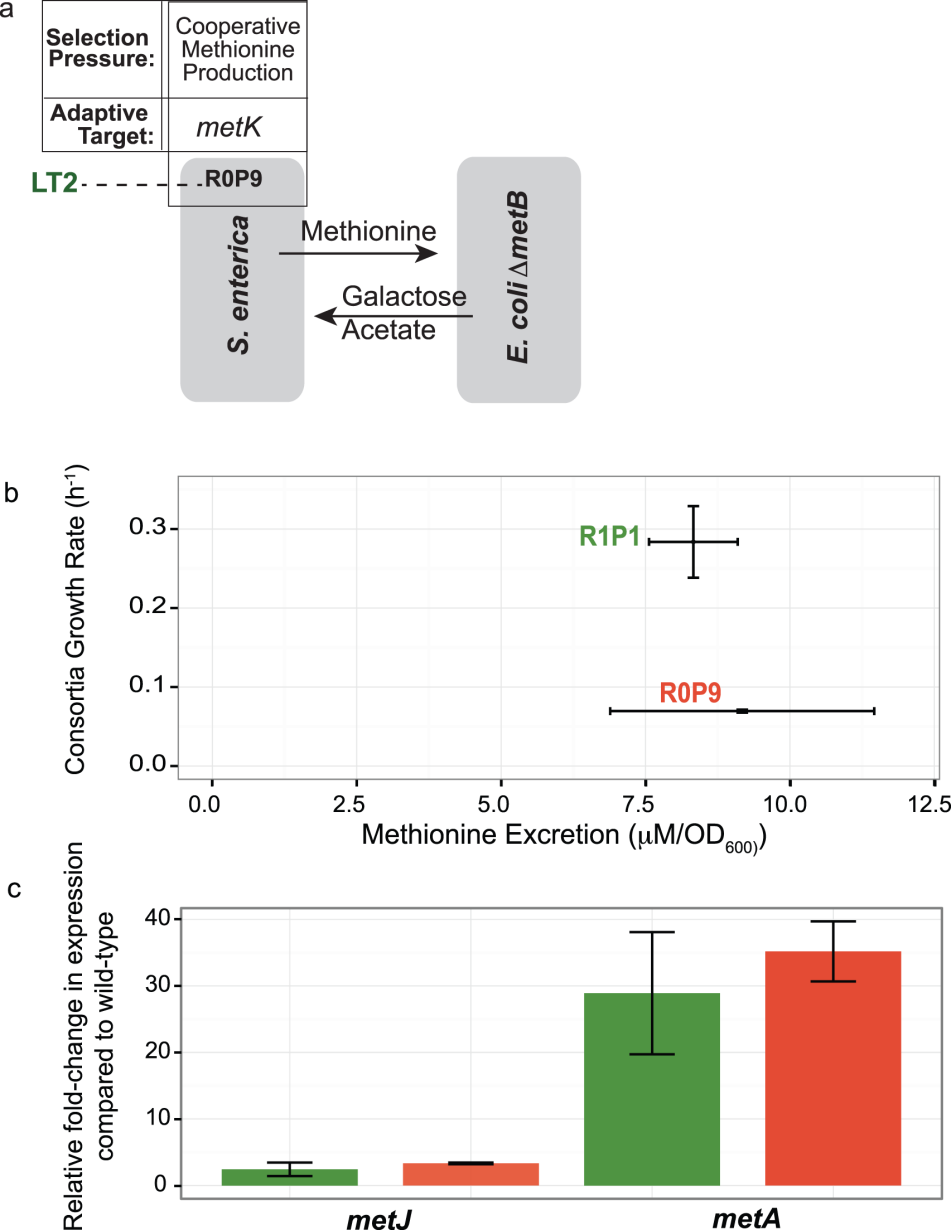
R0P9 evolved cooperation without first selecting for transcriptional deregulation. a) R0P9 was evolved directly from wild-type *S*. *enterica* LT2 co-cultured with an *E*. *coli* methionine auxotroph on lactose minimal media. Sequencing revealed the causative mutation in *metK*, which resulted in methionine production and excretion enabled growth of *E*. *coli* Δ*metB*, which in turn excretes usable carbon for *S*. *enterica*. b) Producer R0P9, evolved without first selecting for ethionine resistance, excretes similar amounts of methionine compared to R1P1, evolved from ethionine resistance background. Yet R0P9, when co-cultured with *E*. *coli* Δ*metB* in lactose minimal media, contributes to a much slower consortia growth compared to R1P1. c) R1P1 and R0P9 express similarly increased levels of *metJ* and *metA* compared to LT2 wild-type ancestor. Error bars indicate standard error of three biological replicates.

We have studied *de novo* evolution of cooperation in a synthetic bacterial consortium where multiple mutational steps were necessary for effective metabolic exchange to occur[11]. The model consortium involves the cross-feeding of metabolites between two mutually-dependent species: an *Escherichia coli* methionine auxotroph (Δ*metB*) and *Salmonella enterica* serovar Typhimurium (Fig 1). When grown in media containing lactose as a substrate and no methionine supplementation, only *E*. *coli* can access the carbon, but only *S*. *enterica* is able to synthesize methionine. *E*. *coli* naturally excretes compounds such as acetate during lactose metabolism, which provides a carbon source for *S*. *enterica*. However, wild-type (WT) *S*. *enterica* LT2 cannot supply sufficient methionine to maintain the *E*. *coli*, and therefore the initial consortia cannot be maintained in lactose minimal media. In the development of this system by Harcombe[11], an *S*. *enterica* that secreted methionine could not be obtained solely though selection for growth in the consortia. Instead, Harcombe used a classic method for generating methionine overproduction: selection for resistance to ethionine (Eth^R^), a toxic methionine analog. Methionine biosynthesis in *S*. *enterica* is tightly regulated via end-product inhibition at the level of both transcription and translation[33–35], and ethionine represses methionine production via the same mechanism[34]. The resulting Eth^R^ strain (R1, for first Resistance mutation) could not support consortia growth either, but in this background it was possible to use selection for consortia growth on solid media to generate a methionine-overproducing strain of *S*. *enterica* (R1P1, also containing the first Production mutation).

In previous work, we characterized the actualizing mutations that permitted methionine production, and thus cooperation in our synthetic consortium; however, we had not identified the potentiating mutation that preceded it[19]. Note two critical differences between the emergence of citrate and the emergence of methionine production by *S*. *enterica*: in the former case, both potentiating and actualizing mutations emerged in the same environmental conditions, and the first citrate utilizers had to rise on frequency over the course of many transfers. In the case of methionine production, this emerged through two successive environments, and in both cases colonies were isolated immediately upon obtaining the desired trait (EthR or consortium growth without methionine). In this study we were explicitly concerned with the epistatic interactions between successive mutations that are required collectively to produce a phenotype, rather than the population dynamics that could have limited its emergence in nature. As such, we used both the original R1 strain and two additional Eth^R^ strains (R2 and R3) as starting points for repeated evolution of methionine excretion in the context of the consortia. This initial selection on solid media gave rise to seven additional, independent cooperative methionine producers that could support community growth in liquid media where performance could be more easily quantified. The stock of *S*. *enterica* we received was a mixture of two closely-related strains, LT2 and 14028s, and the R2 and R3 strains were obtained in the 14028s background, unlike the original R1 strain. Despite subtle genetic differences between these *S*. *enterica* lineages, they are identical in all genes associated with methionine biosynthesis and carbon metabolism[36]. Each of these second step P mutations (P1 to P8) were identified in *metA*, that encodes homoserine trans-succinylase (HTS), the first enzyme of methionine biosynthesis. Molecular modeling suggested that the mutations increased activity of HTS, and hence methionine production, by altering homodimerization. The *metA* mutations were necessary and sufficient for cooperative methionine excretion within the R strains, but were insufficient to generate methionine excretion to support consortia growth when placed alone into their WT backgrounds. The fact that none of the as yet unknown R mutations nor the actualizing P mutations in the WT background could individually recapitulate cooperative levels of methionine excretion demonstrated synergistic epistasis between these loci, but we did not know the identity of the potentiating R mutations.

Here we both attempted to evolve cooperative methionine production directly, and identified and characterized the three actualizing R mutations. Only when we greatly increased the selection time for our original direct selection of WT *S*. *enterica* in a consortium could we obtain a methionine-excreting strain. The responsible adaptive mutation was identified in *metK*, which codes for the enzyme that synthesizes *S*-adenosylmethionine (SAM), a downstream product of the methionine pathway and co-repressor of methionine pathway genes[37,38]. This strain grew quite slowly both alone and in consortia compared to those arising from R backgrounds. The three actualizing R mutations were all in the *metJ* locus, which encodes the methionine pathway repressor, MetJ[39] (Fig 1). MetJ is a transcriptional repressor that, when bound to SAM, inhibits expression of the methionine regulatory pathway[38]. We demonstrate that these *metJ* mutations were necessary for cooperative levels of methionine excretion by *S*. *enterica*, because they reduce or eliminate repression of the methionine pathway. Pairing the previously characterized actualizing *metA* mutations with the potentiating *metJ* mutations was sufficient to generate the fully-cooperative *S*. *enterica* phenotype. The need for a potentiating *metJ* mutation before the actualizing *metA* mutation illuminates the difficulties in overcoming redundant network regulation in the emergence of novel inter-species cooperation.

## Results

### Evolution of cooperation without first selecting for transcriptional derepression is possible, but inefficient

Although our earlier studies evolved *S*. *enterica* cooperators from Eth^R^ backgrounds[11,19], we first readdressed whether direct selection for cooperation with *E*. *coli* Δ*metB* on solid medium could result in discernible cooperation. Six attempts at different times all proved unsuccessful in the experimental timeframe of 3–5 days, so we decided to give a final attempt with a much longer time frame. In this case, *S*. *enterica* LT2 was co-cultured with *E*. *coli* Δ*metB* on agarose plates for eight weeks, with no transfers. Lack of cross-fed nutrients did not result in complete loss of viability of either species, as streaking cell material onto permissive plates for each partner revealed viable cells. Slow leakage of nutrients from cells that were metabolically active but not growing, along with potential impurities in media, could result in growth of a small sub-population sufficient for adaptation, similar to previous systems[40–41]. After eight weeks, a colony formed on the agarose plates, similar to those seen previously from R strains that evolved into cooperators. This colony contained an *S*. *enterica*, termed R0P9 (zero ethionine Resistance mutations, 9^th^ Producer mutation), which excreted enough methionine to support *E*. *coli* Δ*metB* growth (Fig 1).

R0P9 excreted levels of methionine per biomass similar to that of other evolved *S*. *enterica* cooperators like R1P1 (Fig 1; two-tailed Students t-Test, *P* = 0.75). The resulting consortia growth, however, was much slower than any of the strains that emerged from Eth^R^ backgrounds[19] due to the very slow individual growth of R0P9.

### Evolved *metK*^P9^ allele increases transcription of *metA*

Whole genome sequencing of R0P9 revealed a non-synonymous mutation in *metK* [Q120L (CAG→CTG)] as the only mutational difference from ancestral WT *S*. *enterica* LT2, and thus the P mutation for this strain. *metK* encodes the last enzyme in the methionine pathway that catalyzes the formation of SAM from methionine. In *E*. *coli*, SAM inhibits the methionine synthetic pathway at both the transcriptional and translation levels acting as a co-repressor with MetJ[35,38,42,43], and likely behaves quite similarly in *S*. *enterica*[33]. Quantitative RT-PCR data showed that *metA* expression, which is normally repressed by the MetJ-SAM complex, increased by 35.2±9.0 fold in R0P9 relative to WT ancestor, similar to the increased expression in the evolved cooperator R1P1 (Fig 1).

### Mutations in *metJ* occurred in ethionine-resistant strains prior to the evolution of *metA* actualizing mutations

**Fig 2:**
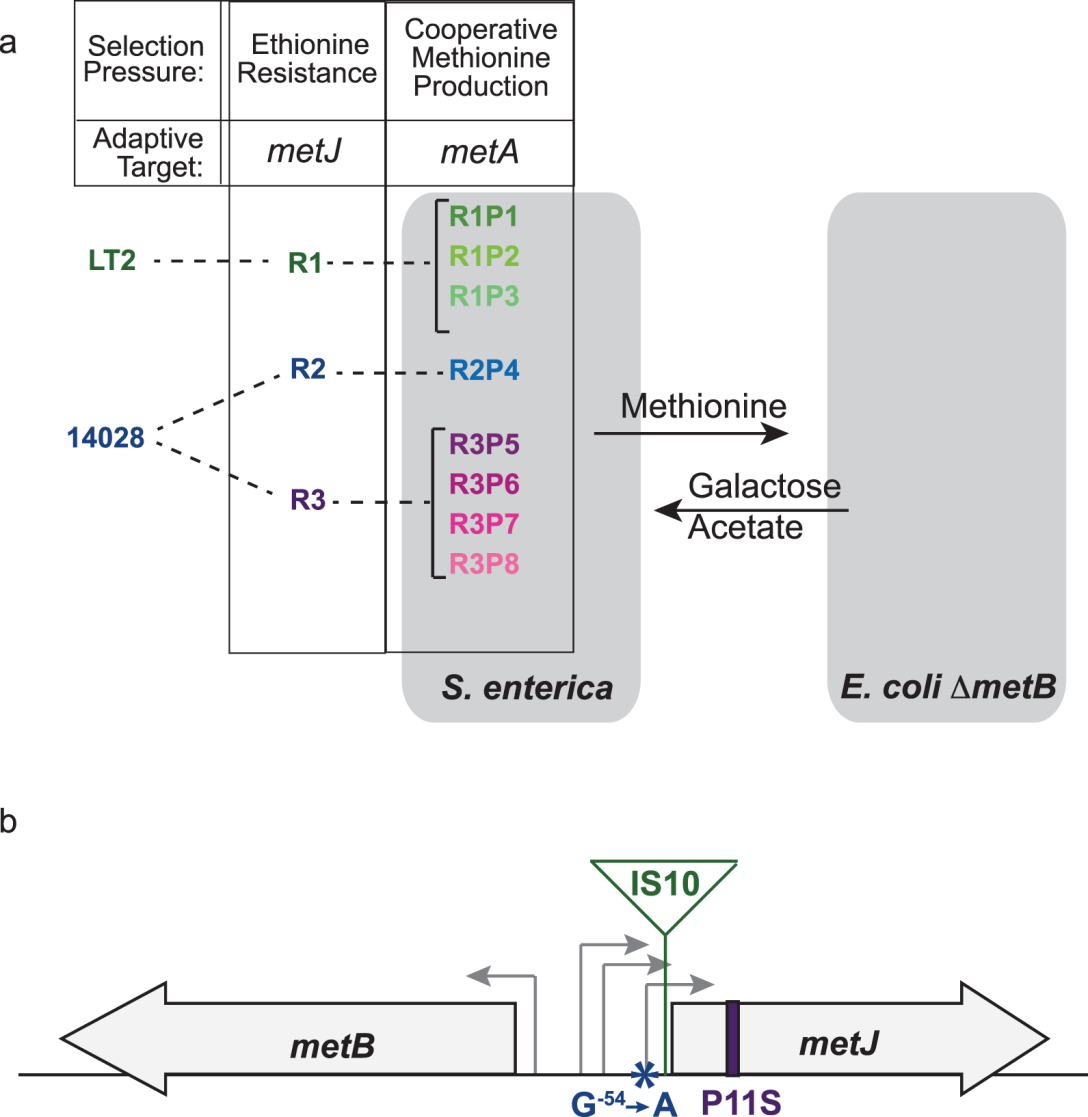
*S*. *enterica* producers evolved from ethionine-resistant strains that feature mutations in *metJ*. a) *S*. *enterica* ethionine resistant strains (R strains) were evolved from wild-type LT2 and 14028s strains, and then co-cultured with *E*. *coli* methionine auxotrophs on lactose minimal media. Adaptive methionine excretion by evolved cooperators enabled growth of *E*. *coli* Δ*metB*. Sequencing revealed parallel mutational targets in each strain during both selection steps: *metJ* during selection for ethionine resistance (detailed in 1b) and *metA* during selection for cooperative methionine production (detailed in [14]). b) A diagram of *metJ* shows the mutations in R1 (IS insertion), R2 (G^-54^➙A mutation in promoter), and R3 (P11S residue substitution) that arose during selection for resistance to ethionine.

To identify the R mutations that we hypothesize potentiated the rapid adaptation of effective cooperation in our earlier studies[11,19], we sequenced the genomes of *S*. *enterica* cooperators R2P4 and R3P5, as well as the ancestral WT strains (Fig 2). The R2P4 sequence differed from WT by five single nucleotide polymorphisms, while R3P5 differed by three. In addition to *metA*, which is the confirmed causative mutation for the second step of our evolution[19], the only other common mutational target was *metJ*, which encodes the methionine operon repressor MetJ (Fig 2). R2P4 contained a G^-54^→A substitution within one of the promoters of *metJ*[44]. R3P5 contained non-synonymous mutation in the *metJ* coding region, P11S. Subsequent targeted sequencing of *metJ* in R1 revealed another mutation at this locus: an IS*10* insertion 16 nucleotides before the translation start site, interrupting the putative ribosome-binding site. Thus, all of our evolved cooperators arose from Eth^R^ strains with mutations in *metJ*. This locus had been identified as a mutational target in the previous screens for Eth^R^[39], and sequencing of *metJ* in the ancestral WT and R strains of *S*. *enterica* confirmed that these mutations occurred during selection for Eth^R^ but before the RP strains evolved cooperative methionine excretion. Note that in all cases there were no apparent phenotypic changes in the *E*. *coli* Δ*metB* partner over this time course.

### *metJ* mutations are necessary to potentiate the actualizing *metA* mutations

**Fig 3:**
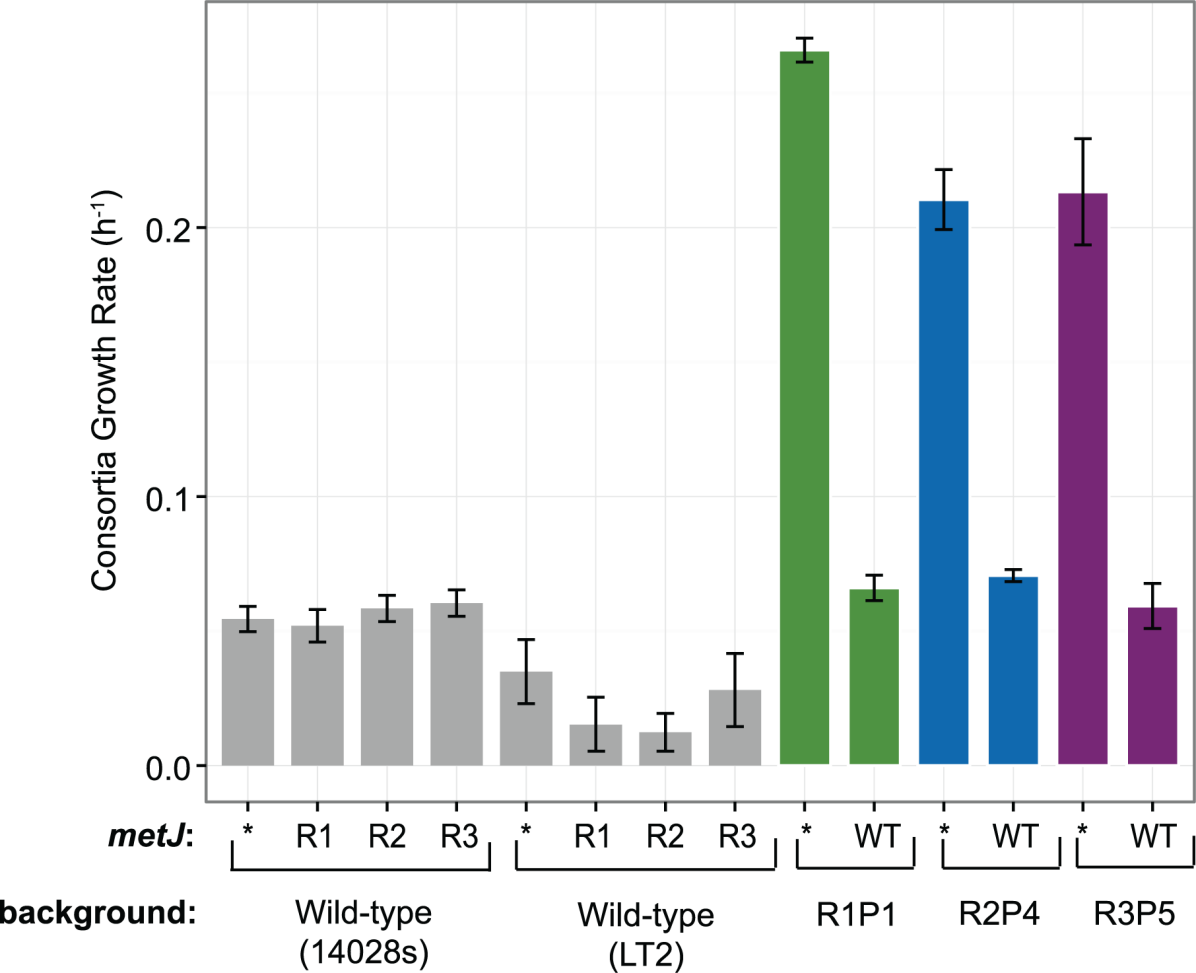
Ethionine resistant *metJ* is necessary for cooperative phenotype in *S*. *enterica*. Wild-type and Eth^R^ alleles of *metJ* were substituted into wild-type and evolved *S*. *enterica* backgrounds. Asterisks indicate no *metJ* substitution. Error bars indicate standard error of three biological replicates.

**Fig 4:**
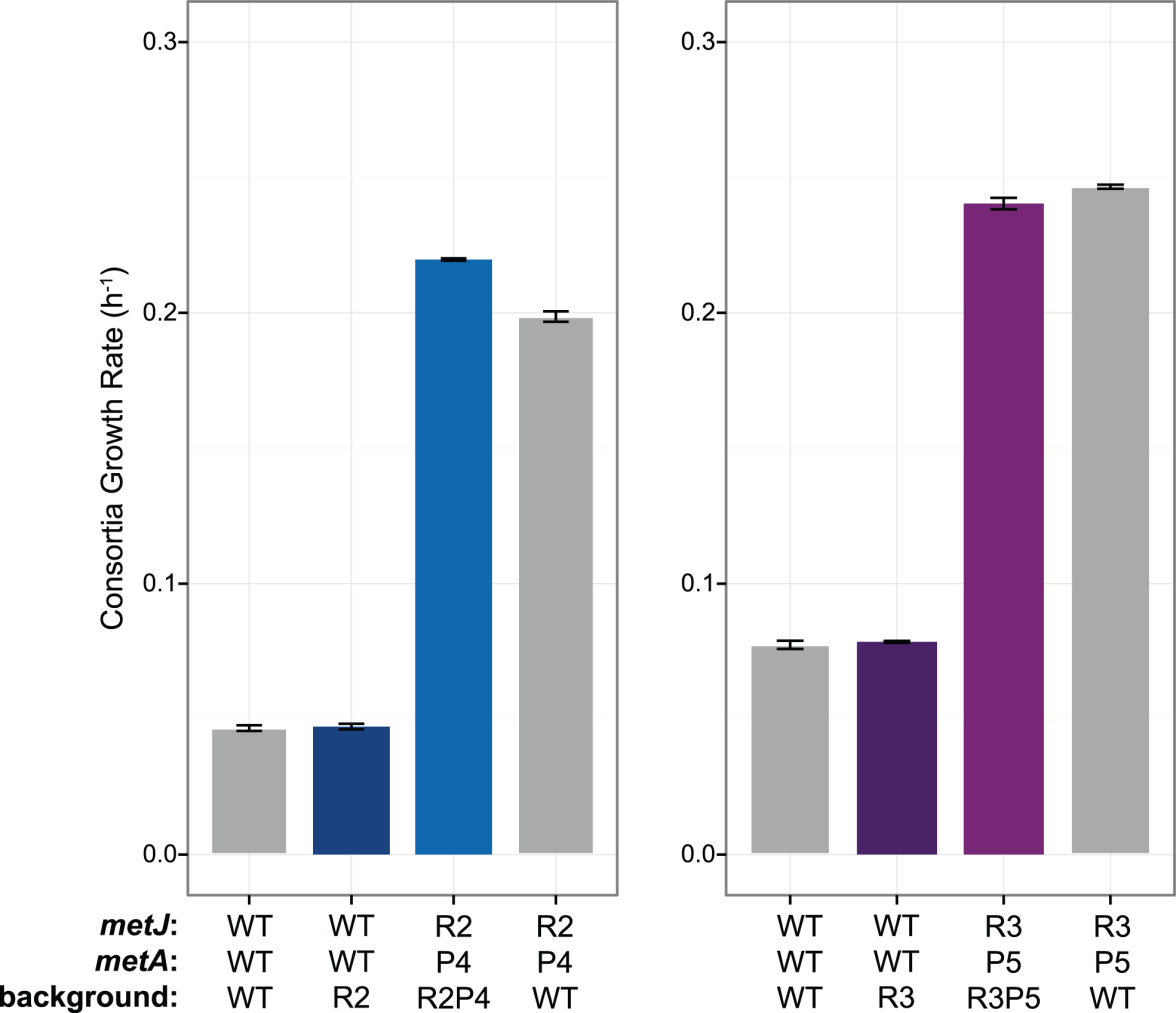
Ethionine resistant *metJ* and evolved methionine producing *metA* alleles are sufficient to recapitulate cooperative behavior in *S*. *enterica*. Error bars represent standard error of three biological replicates. Gray indicates either the original strain, or the reconstructed strain containing the *metA* and *metJ* alleles, such that the rest of their genome is the wild-type background. Differences in wild-type growth rates represent day-to-day variation in growth conditions.

**Fig 5:**
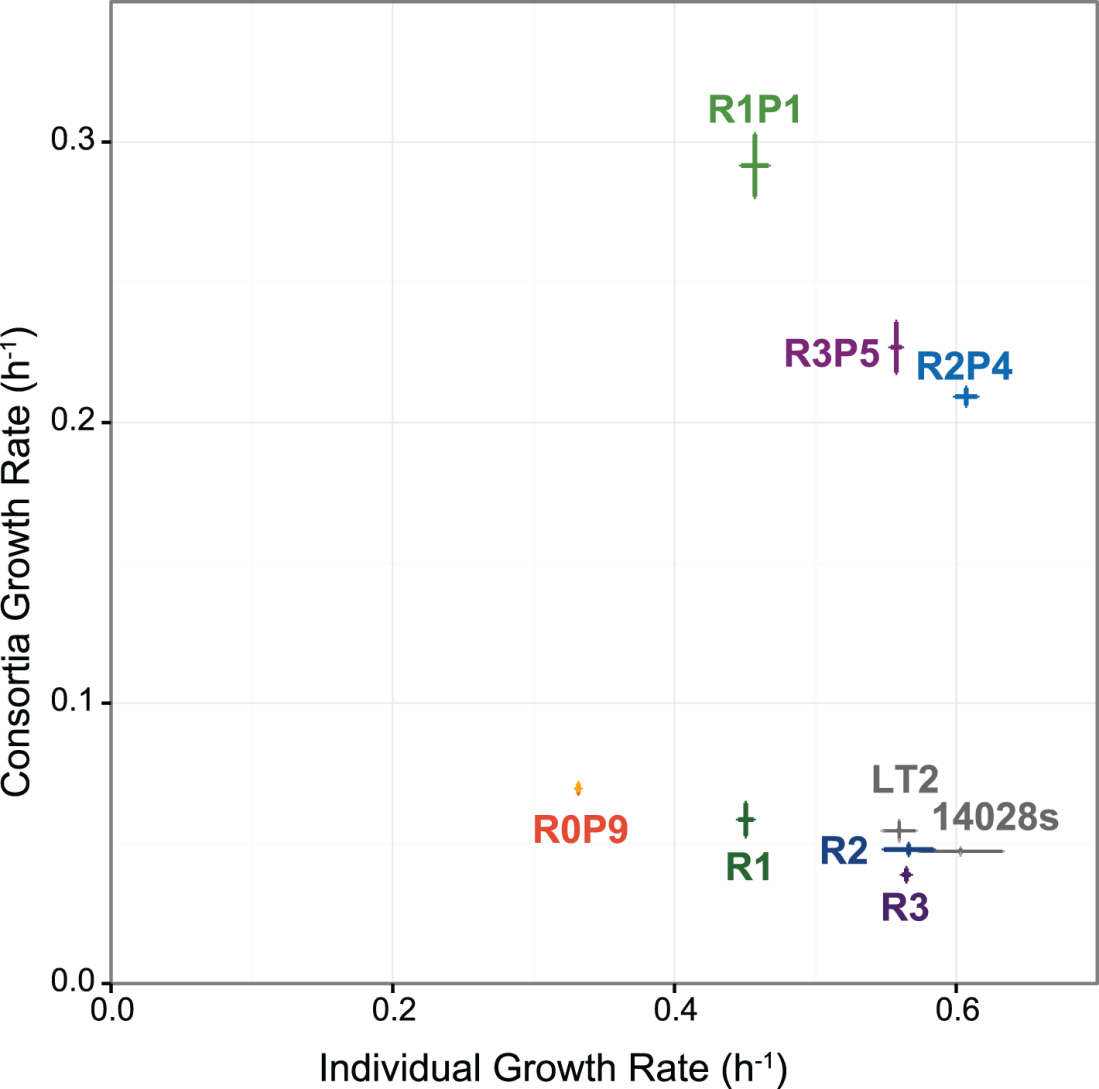
Individual and consortia growth rates for wild-type, ethionine resistant, and evolved strains. Individual growth of *S*. *enterica* strains was measured in galactose minimal media, while consortia growth rates represents *S*. *enterica* strains co-cultured with *E*. *coli* Δ*metB* in lactose minimal media. *S*. *enterica* LT2 is the wild-type ancestor of R1, which evolved into R1P1. *S*. *enterica* 14028s is the wild-type ancestor of R2 and R3, which each evolved into R2P4 and R3P5, respectively. Error bars represent standard error of three biological replicates.

Previously engineered strains with the evolved *metA* alleles substituted into the wild-type background failed to excrete methionine, suggesting that R mutations were necessary for cooperation[19]. Indeed, only insertion of evolved *metA* alleles into R strains enabled methionine excretion. To directly test whether the identified *metJ* mutations were a necessary intermediate step in evolution of cooperation, the ancestral *metJ*^WT^ allele was substituted into cooperator strains and then tested for cooperation in co-culture with *E*. *coli ΔmetB*. Evolved *S*. *enterica* strains with *metJ*^WT^ failed to support cooperative consortia growth, as did wild-type *S*. *enterica* with just *metJ*^R1^, *metJ*^R2^ and *metJ*^R3^ (Fig 3). Only substitution of both evolved *metJ* and *metA* alleles into ancestral backgrounds recapitulated the evolved cooperator phenotype (Fig 4). Thus the *metJ* mutations that arose in the first step of our evolution protocol, along with the *metA* mutations that arose in the second step, are both necessary and, together, sufficient for robust methionine excretion in WT *S*. *enterica*. By comparison, the level of consortia growth provided by R0P9 was greater than R2 and R3 (Fig 5; one tailed Student’s t-Test, *P* = 0.0068 and *P* = 0.00027, respectively), but was insignificantly greater than R1 (one tailed Student’s t-Test, *P* = 0.076). This again highlights how ineffective the one instance of cooperation obtained from direct selection in consortia was compared to the eight strains obtained from the two-step selection procedure.

### Individual-level fitness costs of potentiating *metJ* mutations

To quantify the effects of adaptive *metJ* mutations on *S*. *enterica* growth, individual and consortia growth rates were measured for each WT, R, and evolved RP strain (Fig 5). While the individual growth rate of R2 and R3 in galactose minimal media show a small, but insignificant decrease in growth relative to 14028s (one-tailed Students t-Test, *P* = 0.17 and *P* = 0.14, respectively), both R1 showed a significant decrease relative to LT2 (one-tailed Students t-Test, *P* = 0.00059). Interestingly, the majority of the cost to individual growth incurred by cooperation does not arise from the *metA* mutation that results in methionine excretion, but rather the *metJ* mutations.

### Potentiating *metJ* mutations increase expression of *metA*

**Fig 6:**
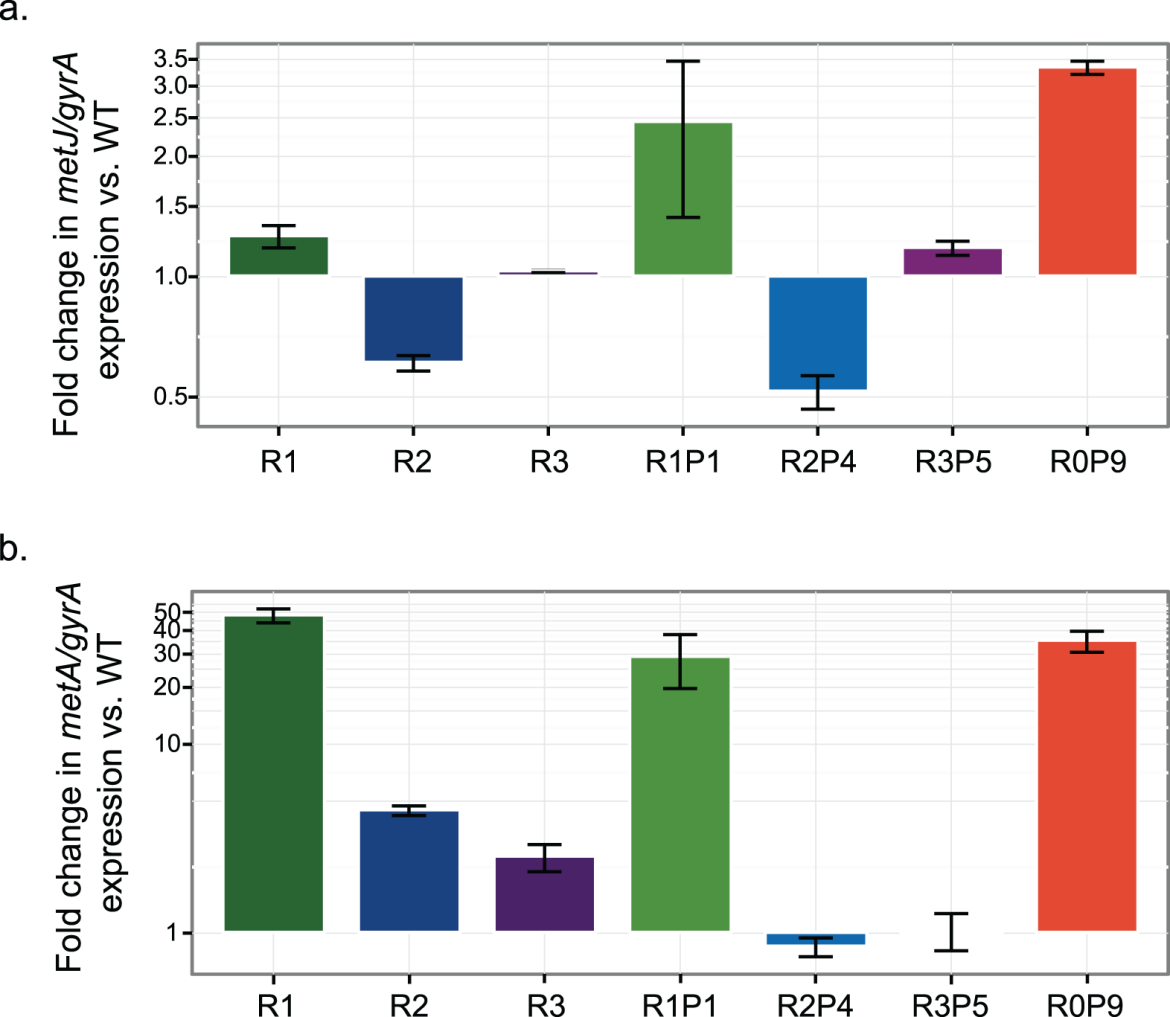
Expression of *metJ* and *metA* in R and RP strains relative to wild-type *S*. *enterica*. mRNA levels were assayed by quantitative RT-PCR for the strains directly obtained due to selection for ethionine resistance or consortia growth in the absence of methionine. Error bars represent standard error of three biological replicates.

Since *metJ* is a transcriptional repressor of most methionine biosynthesis genes, we compared expression *of metJ* and *metA* in R and RP strains to their expression in wild-type (Fig 6, S1 Table). Notably, all *metJ* mutations observed (*metJ*^R1^, IS10 insertion; *metJ*^R2^, G^-54^→A; *metJ*^R3^, P11S substitution in MetJ) yielded minor, less than two-fold changes in *metJ* expression, yet all caused increases in *metA* expression. Despite the R2 mutation being in the *metJ* promoter, and yielding only a modest decrease in *metJ* expression (1.65±0.13 fold decrease), it causes a 4.54±0.52 increase in *metA* expression. In contrast, the R3 mutation is in the MetJ coding region and causes a smaller 2.53±0.83 fold increase in *metA* expression. Given the nature of *metJ*^R3^, we suspect this mutation likely inhibits the function of MetJ, thus decreasing repression of methionine pathway genes. A far greater change in *metA* expression occurred in R1, which exhibited a 48±8.17 fold increase. Although the IS*10* insertion upstream of *metJ*^R1^ generated a 1.26±0.13 increase in *metJ* expression, it transposed into the middle of the ribosome-binding site sequence (5ʹ-AGGAGGA-3ʹ). The high *metA* levels are thus likely due to ineffective translation of the *metJ* transcript. Collectively, these data suggest that all *metJ* mutations work to decrease *metA* repression, but through different mechanisms with varying effects on *metA* expression.

In order to determine whether intracellular methionine exerted any degree of feedback inhibition upon *metA* transcription, we compared *metA* mRNA levels in the R strains vs. the RP strains (Fig 6). For both, R2P4 and R3P5, *metA* levels decreased compared to R2 or R3, demonstrating some remaining responsiveness to end-product inhibition. In contrast *metA* levels were not significantly changed for R1P1 compared to R1, suggesting the *metJ*^R1^ allele was effectively a null allele.

### *metJ* deletion mimics the potentiating R mutations

**Fig 7:**
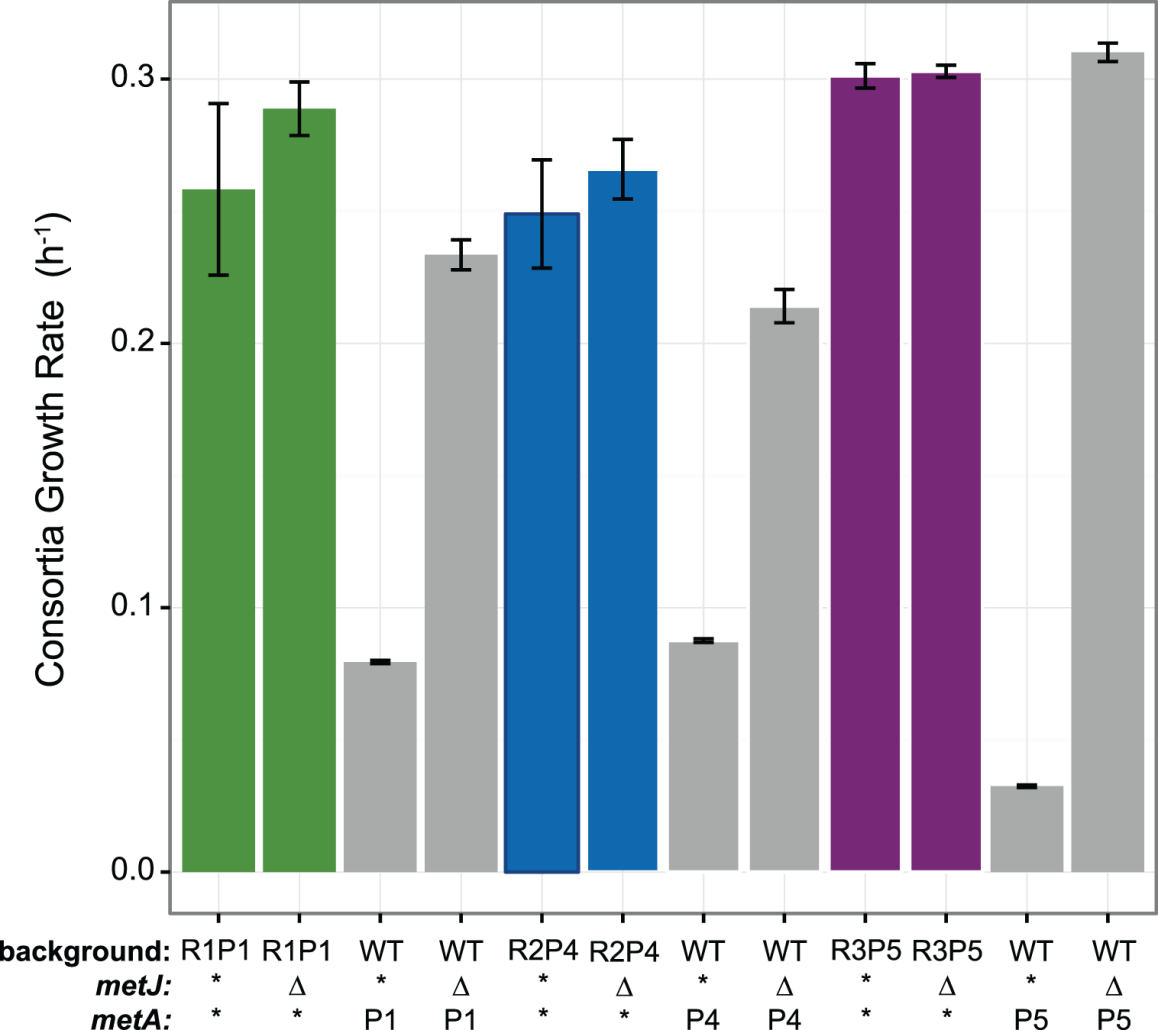
Null *metJ* mutant together with evolved *metA* alleles are sufficient for cooperation in wild-type *S*. *enterica* background. Asterisks indicate native allele (no substitution). Δ*metJ* induced cooperation in wild-type backgrounds containing *metA*^R1P1^, *metA*^R2P4^, *metA*^R3P5^. Gray indicates reconstruction of the indicated *metA* or *metJ* alleles in the wild-type background, whereas the colored bars are the RP strains isolated from selection for consortia growth, or the RP strains with a Δ*metJ* allele.

To directly test how the evolved *metJ* alleles compared to a null allele, we generated a deletion in WT and evolved backgrounds. As with the evolved *metJ* alleles, Δ*metJ* alone does not cause cooperation. When combined with *metA*^P1^, however, Δ*metJ* is sufficient to recapitulate consortia growth in the WT *S*. *enterica* LT2 background (Fig 7). This supports the hypothesis that the R1 mutation in the *metJ* ribosome-binding site results in a null phenotype.

### No specificity in interaction between evolved *metJ* and *metA*

**Fig 8:**
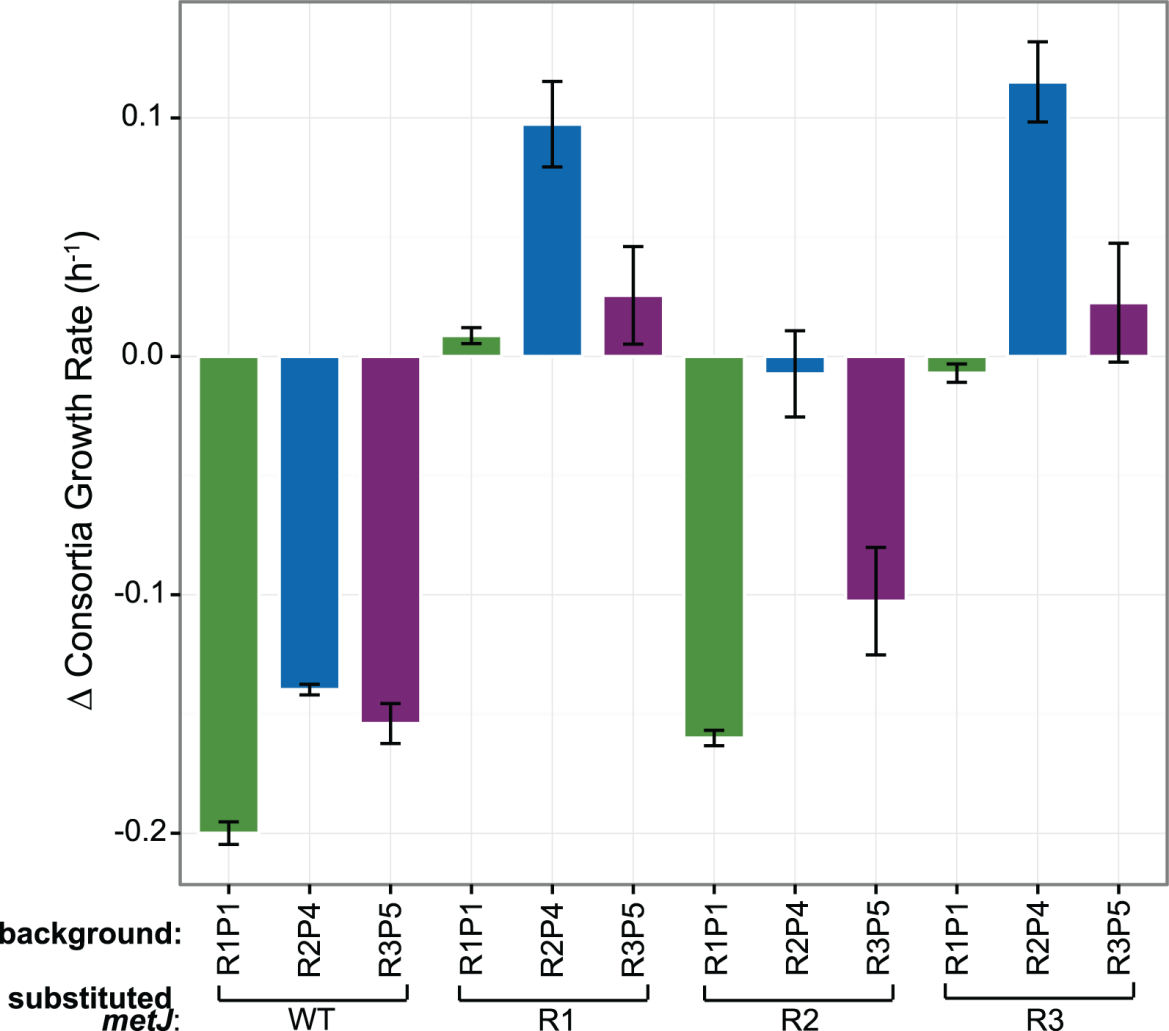
The effect of *metJ* alleles on cooperation in different *S*. *enterica* genetic backgrounds. Mean growth rate of each cooperator with no *metJ* substitution was subtracted from measured consortia growth rates with substituted *metJ*. Error bars represent standard error of three biological replicates.

Given that the *metA* alleles emerged on the background of different *metJ* alleles, we exchanged *metJ* alleles between backgrounds to determine whether there was an specificity in the interactions between particular alleles, as has been shown for other systems[31,32]. To illustrate the differential effects of each *metJ* allele, we calculated the difference in consortia growth rate relative to each corresponding *S*. *enterica* without the allele replacement (Fig 8). *metJ*^R1^ and *metJ*^R3^ have fairly similar impacts on the cooperative phenotype (one-way Tukey's HSD, *P* values ranging from 0.96 to 1), whereby swapping between these alleles having insignificant effects for two of the three sets of comparisons. On the other hand, both cases where *metJ*^R2^ was introduced led to large decreases in growth, and replacing *metJ*^R2^ with either of the other alleles led to fairly similar increases in growth. Examining the effect of the *metA* P alleles, the order P4>P5>P1 was maintained with all *metJ* R alleles tested (although *metA*^P5^ was only significantly better than *metA*^P1^ for the case of *metJ*^R2^). These data indicate that although both individual *metA* and *metJ* alleles vary in their effect on cooperation, there is little evidence of specific interactions between these alleles.

## Discussion

Cooperation through sharing of small, costly cellular commodities can be found in natural microbial populations[5–7]. When shared commodities are costly to produce, explaining the origin of such behavior becomes challenging from both an evolutionary and a mechanistic viewpoint. In particular, how do cooperative genotypes untangle redundant layers of repression that normally regulate the production of precious cellular commodities? Having uncovered the genetic basis of the potentiating (or direct) mutations, we can now address several questions. First, what allowed the *metJ* alleles to potentiate the *metA* actualizing mutations that enabled cooperation? Second, why might these *metJ* alleles have arisen more readily than the *metK* allele? And third, why were the potentiating *metJ* alleles less costly than the *metK* allele?

The basis of potentiation in this system appears to be that the initial selection for Eth^R^ led to loss-of-function alleles in the transcriptional repressor *metJ* that resulted in increased expression of methionine pathway genes, like the actualization target, *metA*. For *metJ*^R1^, the large increase in *metA* transcription (nearly 50-fold) and the insensitivity of *metA* transcription to the high methionine production caused by *metA*^*P1*^ suggest that *metJ*^R1^ was essentially a complete loss of production of the repressor protein. On the other hand, the moderate increase in *metA* transcription (2.5 to 4.5-fold) and the extent of *metA* transcriptional repression due to *metA* P alleles suggest that each of the *metJ*^R2^ and *metJ*^R3^ alleles retained enough MetJ activity to have only a partial derepression. From this background, actualizing mutations arose in *metA* that increased the activity of the biosynthetic enzyme HTS[19]. Individually, mutations in either *metJ* or *metA* are unable to overcome repression at the other level of regulation, and correspondingly neither allele alone leads to effective growth in consortia. Together, they are able to increase the capacity to generate methionine and partially or completely decouple this rate from feedback by cellular methionine concentrations. This temporal ordering where transcriptional regulation is targeted first is a common finding in metabolic engineering, and the evolution of enzymes[45,46]. Though examples do exist where changes in coding sequence proceeded changes in expression in evolving populations[47], regulatory mutants generally precede structural ones in enzyme evolution, especially if the enzyme is very efficient[48].

The *metJ* R alleles and the *metK*^P9^ allele both led to similar levels of *metA* over-expression; why then did Eth^R^ emerge over the course of days, whereas the one successful case of direct selection for cooperation took 8 weeks? As mentioned above, the evolved *metJ* alleles all had partial or complete loss-of-function mutations, and even a Δ*metJ* allele could suffice. Because of this, many changes within the gene due to either point mutations or insertion sequence interruptions could generate this phenotype. On the other hand, the *metK*^P9^ allele likely acts via changing the concentration of SAM in the cell. This creates a balance between needing sufficiently high SAM to supply enzymes needed for methyl transfers throughout biosynthesis, and keeping the levels sufficiently low to not repress methionine synthesis at both the transcriptional and translational levels[49]. It is thus likely that there exists a much smaller mutational target to render MetK less active by the right amount compared to simply needing to destroy MetJ regulation.

Pleiotropy also appears to be at the heart of why the R0P9 lineage with the *metK*^P9^ allele grows more slowly than either the R strains with evolved *metJ* alleles or the RP strains that also contain the evolved *metA* alleles. Loss of MetJ-mediated repression and an increase in MetA activity affect methionine production, but do not otherwise impact cellular physiology. The same cannot be said for affecting methionine synthesis via the concentration of SAM. It will be interesting to learn whether a similar tradeoff exists for evolved deregulation of other biosynthetic pathways: one-step solutions that exhibit broad pleiotropic effects, versus multi-step solutions that may be harder to evolve on their own but lead to a more targeted, insulated effect upon just the pathway in question. Given that end-product inhibition is nearly universal for amino acids and vitamin biosynthesis[49], this process of breaking of regulation via multiple interacting mutations may be a common challenge for genotypes to evolve novel costly cooperation with a partner organism.

The joint requirement for a potentiating and actualizing mutation to achieve discernible cooperation highlights the challenge epistasis can pose upon evolving overproduction and excretion of metabolites whose production is tightly regulated. Epistasis exacerbates the challenges of selecting for cooperation[50], by reducing the abundance of genotypes on which selection can act. As the R mutations were not beneficial on their own in the environment, they would only exist at low levels due to mutation-selection balance. They may be enriched in an environment such as one containing ethionine where derepression is beneficial, but it is unclear whether such conditions exist in nature. Conversely, sufficiently small population sizes, such as may be created upon inoculation of a new environment by one or a few cells, could also allow the deleterious derepression of methionine to rise in frequency due to drift. In this regard, it is perhaps unsurprising how commonly intracellular bacteria have entered into mutualisms involving nutrient exchange[9,51]. The bottlenecks of vertical inheritance would readily allow the deleterious effects of partial derepresssion to persist, thereby potentiating the further actualizing mutations that create the overproduction of nutrients to emerge and be selected upon at the level of the holobiont.

## Methods and materials

### Growth media and strains

The experimental system consisted of an *E*. *coli* methionine auxotroph (*E*. *coli* Δ*metB*) and an ethionine-resistant *S*. *enterica* partner. The *E*. *coli* strain K12 BW25113 with a *metB* knockout was obtained from the Keio collection, with the only modification being that it had its lactose metabolism restored as described[11]. Ethionine-resistant *S*. *enterica* mutants from LT2 and 14028s backgrounds were selected as described[11]. Cultures were grown in “Hypho” minimal media containing trace metal mix[32] and were supplemented with either 0.1% (liquid media) or 0.05% (solid media) galactose or lactose. Consortia were initiated by growing each partner alone in medium sufficient for their nutritional needs (*E*. *coli* on lactose minimal media with methionine added; *S*. *enterica* on glucose minimal media), and these would then be added as equal volumes of dense overnight cultures. Antibiotic concentrations used were: 50 μg/mL ampicillin, 25 μg/mL chloramphenicol. All antibiotics and chemicals obtain from Sigma Aldrich (St. Louis, MO) unless otherwise noted. All data are available in S2 Table, and all strains used are listed in S3 Table.

### Evolution of methionine excreting *S*. *enterica* mutants

The two-step selection process for evolving a methionine-excreting *S*. *enterica* LT2 mutant occurred as described[11]. Briefly, initial selection on ethionine, a competitive methionine analog, was utilized to create new evolutionary starting points in *S*. *enterica* serovar Typhimurium 14028s. Co-culturing of ethionine-resistant *S*. *enterica* and *E*. *coli* Δ*metB* on lactose minimal media agarose plates to select for methionine excretion proceeded as described[11,19]. Evolution of cooperator R0P9 arose from co-culture of 500 μL of *S*. *enterica* LT2 grown to saturation in Hypho glucose and 500 μL of *E*. *coli* Δ*metB* grown in Hypho lactose with 100 μM methionine, pelleted, resuspended in minimal media, spread onto a lactose Hypho agarose plate, and transferred after 8 weeks of growth at 30°C to isolate new cooperative colony at a 1:10 dilution.

### Genomic sequencing

Genomic DNA from *S*. *enterica* wild-type strains LT2 and 14028s, and our evolved *S*. *enterica* strains R3, R2P4, R3P5, and R0P9 was extracted from lysed cells via phenol chloroform extraction[52], and prepared for Illumina sequencing using TrueSeq kit. Samples were sent to The Microarray and Genomic Analysis Core facility at the University of Utah for sequencing on Illumina HiSeq 2000 sequencer, and aligned and analyzed using breseq, with all default users settings other than enabled polymorphism prediction [53] (http://www.barricklab.org/breseq). DNA sequencing reads are deposited in the NCBI SRA.

### Gene disruption and plasmids utilized

Deletion of *metJ*-*metB* were performed using the method of Datsenko and Wanner[54] with modifications described by Ellermeier *et al*.[55]. A selectable chloramphenicol marker (*cat*) flanked by 40 bp of the region surrounding the coding region of *metJ-metB* was constructed via PCR using plasmid pKD32 as template[54]. PCR product was cleaned using QiaQuick PCR Purification kit (Qiagen, Valenica, CA) and electroporated into electrocompetent *S*. *enterica* cells carrying lambda Red helper plasmid pKD46[54]. Cells were suspended in LB and recovered for 1 hr shaking at 37°C before being spread on selective media. Cells were purified once more selectively at 37°C before Δ*metJB*::*cat* insertion was verified via PCR.

### P22 transduction

To create lysates for P22 transduction, *S*. *enterica* donor strains were grown overnight, and then diluted 1:500 in 5 mL LB+cat with 150 μL P22 HT *int* lysate stock and grown with shaking at 37°C for approximately 6 hours. After vortexing with 1 mL chloroform to kill remaining donor cells and centrifuging 10 minutes at 4550 x *g* to remove debris, lysate was stored at 4°C for up to 3 years. 200 μL overμnight culture of recipient *S*. *enterica* strains were incubated with 100 μL lysate for 25 minutes at room temperature, rinsed twice with 100 mM sodium citrate LB, plated onto selective media, and grown overnight. After purifying once more selectively at 37°C, strains were cross-streaked against lytic P22 H5 lysate to test for remaining presence of phage.

### Allele replacement

Native *metJ/metB* loci were deleted via P22 transduction of Δ*metJ/metB*::*cat* and selection on LB+chloramphenicol. Cured Δ*metJ/metB* strains received replacement loci via P22 transduction of donor strains containing the desired new *metJ/metB* locus and selection on glucose minimal media. *metB*, located within the 1.4 kb region downstream of *metJ*, was included in this deletion to allow growth in minimal media to be used as a counter-selection against allele replacement. *metJ* is not necessary for growth in these conditions, and Δ*metJ* strains would be indistinguishable from successful transformants in this selection.

### Quantitative RT-PCR

10 mL cultures were grown to early log phase (OD_600_ = 0.10–0.12) in galactose minimal media, and pellets were snap frozen in liquid nitrogen before storing at -80°C. Cells were lysed and RNA was extracted using an RNAeasy Kit (Qiagen) according to the manufacturer’s instructions. Reverse transcription was performed using SuperScript III Reverse Transcriptase (Invitrogen, Carlsbad, CA) according to manufacturer’s instructions, using gene specific primers. qPCR was performed using fast EvaGreen PCR Master Mix (Biotium, Hayward, CA) and quantified on a CFX384 Touch Real-Time PCR Detection System (BioRad, Hercules, CA). *metA* and *metJ* gene expression values for each strain represent three biological replicates, each of which is composed of three technical replicates. Relative quantification proceeded as described[56], using *gyrB* as reference gene. Standard curves for each primer set, as well as no reverse transcriptase and no template controls, were included on the same plate as experimental samples.

### Methionine measurements

Methionine measurements via GC-MS closely followed the method of Zamboni *et al*.[57], and are detailed for this system in [19].

## Supporting information

S1 Table Q-RT-PCR data of *metJ* and *metA* expression. (XLSX)

S2 Table Underlying data from figures. (XLSX)

S3 Table List of strains used. (XLSX)
